# Pan-genomic characterization and structural variant analysis reveal insights into spore development and species diversity in Ganoderma

**DOI:** 10.1099/mgen.0.001328

**Published:** 2024-11-20

**Authors:** Hang Yu, Shasha Wang, Lina Wang, Weixin Wu, Wei Xu, Shuisheng Wu, Xiaoyan Li, Wen Xu, Zehao Huang, Yu Lin, Haifeng Wang

**Affiliations:** 1 Innovation and Transformation Center of Science and Technology, College of Pharmacy, Fujian University of Traditional Chinese Medicine, Fuzhou, Fujian 350122, PR China; 2 State Key Laboratory for Conservation and Utilization of Subtropical Agro-Bioresources, Guangxi Key Lab of Sugarcane Biology, College of Agriculture, Guangxi University, Nanning, Guangxi 530005, PR China; 3 Key Laboratory of Crop Cultivation and Physiology, Education Department of Guangxi Zhuang Autonomous Region, Guangxi University, Nanning 530004, PR China

**Keywords:** *Ganoderma*, pan-genome, structural variation, MSH4, spore development

## Abstract

Understanding the genomic diversity and functional implications of *Ganoderma* species is crucial for elucidating their evolutionary history and biotechnological potential. Here, we present the first pan-genomic analysis of *Ganoderma* spp., combining five newly sequenced genomes with ten publicly available genomes. Our comprehensive comparative study unveiled a rich genomic landscape, identifying core genes shared among all *Ganoderma* strains and species-specific gene sets. Additionally, we identified structural variants impacting the expression of key genes, including insights into the *MSH4* gene involved in DNA repair and recombination processes, which exhibits a 440 bp insertion in the promoter region and a leucine-to-serine mutation in the gene body, potentially increasing spore production in the S3 strain. Overall, our study provides valuable insights into the genomic architecture and functional diversity of *Ganoderma*, paving the way for further research on its evolutionary dynamics, biotechnological applications and pharmaceutical potential.

**Impact Statement**

Using the third-generation sequencing technology, we assembled five genomes of *Ganoderma* strains and integrated them with ten published reference genomes, making the first pan-genomics endeavour for *Ganoderma* spp. Our study delineated core genes and species-specific genes across these strains, unveiling specific structural variants influencing the gene expression of *MSH4*, which is involved in DNA repair and genetic recombination processes. Our findings shed light on the regulation of *Ganoderma* spore development, offering invaluable insights into its biological properties, discovery of bioactive components, genetic improvement, breeding of superior varieties and understanding of the evolutionary history.

## Data Availability

The raw sequencing reads generated in this study have been deposited in the National Center for Biotechnology Information sequence read archive (SRA, https://www.ncbi.nlm.nih.gov/sra) with the BioProject accession number: PRJNA992876. The sample names and corresponding access numbers have been deposited in Table S20. The genome file has been deposited in the CNCB Genome Warehouse (GWH, https://ngdc.cncb.ac.cn/gwh) under accession number GWHDTXQ00000000 for S1, GWHDTXR00000000 for S2, GWHDTXP00000000 for S3, GWHDTXO00000000 for S4 and GWHDTXN00000000 for S6. The annotation files for the genomes are stored in the Figshare database at the following link https://doi.org/10.6084/m9.figshare.23650680.v1.

## Introduction

*Ganoderma lucidum*, which is commonly known as Lingzhi or Reishi, is a large fungus valued for its medicinal and edible properties. It belongs to the Ganodermataceae family, has a long history of use in traditional Chinese medicine and is included in pharmacopoeias [1]. Lingzhi consists of three parts: mycelium, basidioma and fruiting body. Lingzhi spores, which are small reproductive cells ejected from the mature fruiting body during its growth stage, contain all of the active substances found in Lingzhi, including polysaccharides, ganoderic acids, organic germanium and organic selenium [2]. Lingzhi, especially Lingzhi spore powder, is widely used for its antitumour activity, immune enhancement, liver protection, anti-inflammation, blood sugar reduction and antioxidation [3,4]. It is extensively employed in health products and immune regulators and as an adjunct to cancer treatment, holding significant research value and application prospects. Lingzhi is widely grown in China. It has numerous varieties, each of which exhibits distinct morphologies and textures. Although the genomes of more than a dozen varieties of *Ganoderma* have been assembled [5,6], different *Ganoderma* have different properties and medicinal effects; thus, it is necessary to sequence the genomes of additional *Ganoderma* varieties.

**Fig. 1.**
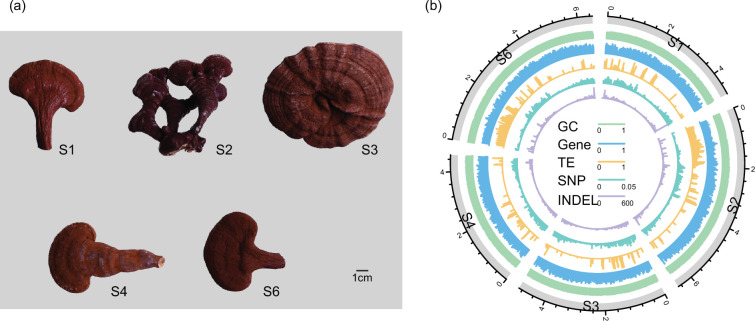
Five XZL *Ganoderma* varieties and genome overview. (**a**) Fruiting bodies of the five XZL *Ganoderma* varieties. (**b**) Circos plot illustrating the chromosome 3 annotation across the S1–S6 genome assembly. The genome sequence was segmented into 20 kb windows for analysis.

Xianzhilou (XZL), a highly valued native medicinal herb in Fujian Province, China, boasts a rich assortment of varieties and is extensively cultivated. It plays a central role in Fujian’s traditional Chinese medicine industry and is a vital source of income for local farmers. Among these varieties, the five XZL varieties (XZL S1, known for high sporulation yield; XZL S2, cultivated from Songshan Lingzhi; XZL S3, a novel variety; XZL S4, with a high fruiting body yield and XZL S6, possessing both sporulation and fruiting yields) (Fig. 1a) occupy the largest cultivation area and highest economic value. XZL, as a *Ganoderma* variety with excellent characteristics, can support the selection and breeding of improved *Ganoderma* varieties by studying the genomic differences between it and other *Ganoderma* varieties, as well as by performing pan-genomic and other studies.

Structural variation (SV) is a prominent driver of genetic evolution within genomes, influencing species diversity [7]. These SVs, such as deletions, insertions, duplications, inversions and translocations, play a pivotal role in species evolution, notably in the development and growth of crops. Their effects ripple through critical aspects, such as tissue structure, flowering time, fruit size and resistance traits [8], thereby contributing to the genetic diversity and adaptability of crop species. Hence, accurately identifying genome SVs is a prerequisite for unravelling species diversity and evolution. Advancements in third-generation sequencing have enhanced the quality of genome assembly; furthermore, large-scale population resequencing and genetic studies now facilitate the precise identification of SVs in a reference genome. However, relying solely on a single reference genome has limitations. Diverse varieties within the same species often exhibit substantial genomic disparities. For example, soybean resequencing analysis revealed that 4448 material-specific genomic segments were absent from the reference genome, with 1148 segments exceeding 500 bp in length [9]. This highlights the fact that, as a single reference genome, the genetic heterogeneity among different individuals’ genomes merely represents one individual and cannot encompass the entirety of genetic variation within a species. The field of pan-genome studies seeks to overcome the limitations intrinsic to a single reference genome [10]. Pan-genomics consolidates genome data from multiple individuals within the same species, forging a comprehensive repository that endeavours to encompass as many species’ genome sequences and information as possible. This approach has been widely applied in major crops, such as soybean [11], maize [12], tomato [13], rice [14] and barley [15], revealing core and unique genes among varieties and discerning specific SVs. This all-encompassing strategy precisely reveals genetic diversity within species, propelling research into species evolution and functional genomics while bequeathing valuable and ample genetic resources for species enhancement [16].

To comprehensively unveil the genomic diversity and unearth widespread SVs in these five XZL varieties, this study integrated the published Lingzhi genomes and conducted whole-genome sequencing of the five XZL varieties for pan-genome analysis. In order to identify core genes in Lingzhi and crucial SVs among the XZL varieties, we assembled and annotated the genomes of these five XZL varieties with high-quality *de novo* sequencing, determined their phylogenetic relationships and evolutionary history and employed pan-genome analysis in conjunction with ten other published Lingzhi genomes [6]. Our findings show that SVs lead to differential gene expression, affecting significant Lingzhi phenotypes. Furthermore, we identified key genes influencing spore synthesis and triterpene compound metabolism pathways. In addition to offering essential data support for the genetic diversity of Lingzhi, our research provides valuable resources for cultivating superior Lingzhi varieties and promoting the development of the traditional Chinese medicine industry.

## Methods

### *Ganoderma* materials

Five *G. lucidum* samples (S1, S2, S3, S4 and S6) were provided by Xianzhi Technology (Fujian, China) Co., Ltd. Forty fresh * G. lucidum* samples (four each of mature fruiting bodies and dikaryotic mycelium of S1, S2, S3, S4 and S6) were cultured for 7 days at 28 °C on potato dextrose agar (PDA) (medium: potato 200 g, dextrose 20 g, agar 20 g and deionized water 1 l) and the relative humidity was set at 70–80%. The strains were collected after mycelia had colonized the entire plate, and then DNA was extracted.

### Illumina sequencing

QC-qualified DNA was ultrasonically fragmented into target fragments, and the Illumina library was obtained through fragment purification, end repair, addition of 3'A tails, ligation of junctions and PCR enrichment. Bipartite sequencing was then performed using a MinION sequencing analyzer (Oxfordshire, England).

### PacBio sequencing

After passing the quality inspection, the DNA was randomly interrupted, and 1* magnetic beads were added to purify the DNA. DNA damage and end repair were performed, and barcode labels and sequencing junctions were made at both ends of the DNA fragments. The library was then constructed and sequenced.

### Hi-C sequencing

Hi-C sequencing was performed on five XZL strains of *Ganoderma*. After fixation with formaldehyde, the cells were lysed to produce sticky ends and ligated with ligase through biotin labelling. The DNA was then randomly broken, generating the target fragments for library sequencing. To ensure the validity of the subsequent analysis, it was necessary to filter the Hi-C sequencing data. Data containing splice sequence reads, having a consecutive quality of less than 20 bases, or having a read length <50 bp were filtered. The paired reads were retained, and Hi-C library construction was performed.

### *Ganoderma* RNA extraction and sequencing

The total RNA of 40 fresh *Ganoderma* samples (four biological replicates each of *Ganoderma* mycelium and fruiting bodies for S1, S2, S3, S4 and S6) was extracted by the TRIzol method, and the quality of the extracted RNAs was examined by the ultramicro spectrophotometer, agarose gel electrophoresis and other instruments, and commissioned to the BGI-shenzhen Limited for sequencing and analysis by using the MGISEQ-2000.

### Genome estimation, assembly and validation

We used *k*-mer analysis (k=21) for the estimation of the size of the *Ganoderma* genomes, which was calculated by Jellyfish (v2.2.10, -m 21 s 10M) [17], and the distribution was displayed and analysed using the software GenomeScope (v2.0) [18]. Five XZL genomes were assembled based on long reads obtained from PacBio sequencing and Hi-C data using the default parameters of Hifiasm (v0.16.1-r375) [19]. Redundant sequences in contigs were removed using Purge_haplotigs (v1.1.2, -l 10 m 130 h 200) [20]. The obtained de-redundant contigs were mounted using Ragtag (v2.1.0) [21]. Pilon (v1.24) [22] was then used twice to improve genome accuracy based on second-generation Illumina short reads sequences. For the evaluation of genome completeness, the BUSCO (v5.2.2, fungi_odb10) [23] evaluation methods were used, and Illumina short reads were mapped to the assembled genome with BWA (v0.7.17) software [24].

### Genome annotation

We used RepeatModeler (v2.0.1) [25] to identify repetitive sequences in each XZL *Ganoderma* combined with Repbase library (https://www.girinst.org/repbase/, v29.09) and then used RepeatMasker (v4.0.9) [26] for reference genome matching to obtain annotation files of repetitive sequences. The five XZL genomes were genetically annotated using a combination of RNA-seq data, homology sequence and *de novo* prediction methods. RNA-seq data were obtained from the mycelium and fruiting body of the respective XZL. Homologous gene sequences were collected from the *Ganoderma* genomes published by Jiang *et al*. [6]. These data were used to build the final gene model through the GETA (https://github.com/chenlianfu/geta) pipeline. The eggNOG online software (http://eggnog-mapper.embl.de) [27] was used for the functional annotation of genes. Specific codes for genome assembly and annotation can be found at the following link: https://github.com/yuhang5810/Genome-assembly-for-Ganoderma.

### Gene family identification and phylogenetic analysis

Gene families were analysed for 24 fungal species using the default parameters of OrthoFinder (v2.5.1 -M msa -T raxm) [28]. Maximum-likelihood phylogenetic trees were constructed using RAxML-NG (v1.1.0) [29] after matching single-copy genes in gene families using muscle (v3.8.1551) [30]. R8s (v1.81, -b -f) [31] was used to predict the divergence time of 24 species. We used CAFE (v4.0) [32] to obtain the number of contractions and expansions of gene families in the phylogenetic trees of 24 species using the output of OrthoFinder as input. For the classification of gene families, we classified them into four distinct categories. Among these, core families, encompassing genes, present in all 15 species. Families identified in 13–14 species were designated as softcore families. Families identified in 2–12 species were designated as dispensable families. Finally, gene families detected in only one species were catalogued as private gene families.

### Genomic structure analysis

SYRI (v1.5) [33] software was used to identify SVs among the XZL genomes. SnpEff (v5.1d) [34] was used to recognize the degree of effect of SVs on genes, with the degree categorized as follows: https://pcingola.github.io/SnpEff/se_inputoutput/.

### RNA-seq analysis

For the raw transcriptome data, we used Trimmomatic (v0.39) [35] to remove adapters and low-quality sequences and then used HISAT2 (v2.2.1) [36] to align to the reference genome. Gene expression levels were quantified using StingTie (v2.1.4, -e -B) [37]. DESeq2 (v1.44.0) [38] was used to calculate differentially expressed genes (DEGs), and for the definition of DEGs, we chose log_2_|Fold Change|>1 and false discovery rates <0.05.

### Gene ontology enrichment analysis

For gene ontology (GO) enrichment analysis of key genes, we used GOATOOLS (v1.0.15, --pval=0.05) [39] to perform that only GO terms with *P*-value <0.05 were extracted and used for subsequent analysis.

### Spore collection and measurement

The five XZL strains were cultivated from basswood, with their fruiting bodies maturing in ~70 days at an incubation temperature of 20–25 °C. When the *Ganoderma* matured, the ground was covered with mulch to isolate the sediments, a membrane garden cylinder was placed around the mushroom cap, and the bag was tied tightly to the sleeve to collect the spore powder. After 60 days, the spores were collected under closed conditions to avoid external impurities. The content of spore in S2 and S4 is too low to collect. A total of six planting sheds were sampled for the experiment. Ten pieces of *Ganoderma* were taken from each shed separately for data counting.

$$The\;ratio\;of\;sporulation\;\left(\%\right)=\frac{Sporulation\;yield\;\left(g\right)}{Dry\;weight\;yield\;of\;the\;fruiting\;body\;\left(g\right)}.$$

## Results

### *Ganoderma* genome sequencing and assembly

This study focused on five varieties of *G. lucidum* native to Fujian Province, China, known as the XZL varieties, denoted as S1, S2, S3, S4 and S6 (Fig. 1a). We initiated the investigation by performing Illumina short-read sequencing, yielding data amounts of 13.4, 13.6, 13.9, 13.9 and 16.3 Gb (262–335X) for S1, S2, S3, S4 and S6, respectively (Table S1, available in the online version of this article). Employing a genome survey analysis method (*K*=21), we estimated the genome sizes for the five varieties, resulting in approximations of 45.1, 45.7, 46.4, 46.8, and 43.8 Mb, respectively. The estimated genome heterozygosity values were 1.6, 1.5, 1.5, 1.5 and 1.6% respectively (Fig. S1, Table 1). These estimations are closely aligned with previously published *Ganoderma* genome sizes and heterozygosity [6].

**Table 1.** Statistics for genome assembly

		S1	S2	S3	S4	S6
Genome sequencing depth (X)	PacBio sequencing	83.3	104.4	86.6	71.5	91.6
	Illumina sequencing	269.3	280.9	276.2	262.5	335.4
	Hi-C	372.0	–	345.7	335.1	–
Estimated genome size, bp		45 131 255	45 673 508	46 351 235	46 805 969	43 763 555
Estimated heterozygosity, %		1.61	1.54	1.53	1.50	1.62
Number of chromosomes		12	12	12	12	12
Total length of scaffolds, bp		49 852 960	48 516 701	50 488 727	53 068 536	48 625 026
Anchored chromosomes, %		97.12	99.34	96.58	97.17	97.10
Scaffolds N50, bp		4 675 681	4 077 397	4 199 539	4 400 400	3 732 749
Longest scaffold, bp		6 185 067	6 509 122	5 991 591	5 504 339	6 495 135
Number of contigs, bp		52	73	56	44	48
Total length of contigs, bp		52 367 691	95 841 500	50 659 871	53 086 883	48 643 294
Contigs N50, bp		3 957 197	3 540 950	3 526 000	4 298 372	3 414 438
GC content %		55.6	55.9	55.7	55.5	55.7
Mapping with Illumina reads, %		97.81	85.99	86.68	98.52	82.14
BUSCO for assembly, %		96.4	96.1	96.3	96.4	96.2
BUSCO for annotation, %		97.3	98.5	99.6	99.3	99.2

To achieve high-quality *Ganoderma* genomes, we employed Hifiasm for the initial assembly using 4.2, 5.1, 4.4, 3.8 and 4.5 Gb (71–104X) of third-generation PacBio HiFi reads (Table S2), followed by error correction using Illumina short reads. Hi-C sequencing data (Fig. S2, Table S3) facilitated anchoring of the genomes to 12 pseudo-chromosomes, resulting in final genome sizes of 49.9, 48.5, 50.5, 53.1 and 48.6 Mb, all with mount rates exceeding 96% (Table 1). We evaluated genome quality using the BUSCO method, with all five genomes exhibiting completeness levels exceeding 96%. Together, these findings indicate that we have successfully assembled five high-quality *Ganoderma* genomes.

### Genome annotation of five XZL genomes

We carried out comprehensive annotations on the five XZL genomes, namely S1, S2, S3, S4 and S6. Notably, the largest genome, S4, boasted the highest count of coding protein genes at 14 779, while the number of protein-coding genes in the other four strains was 14 140 (S1), 13 897 (S2), 14 362 (S3) and 13 959 (S6). The average gene length across these five genomes ranged from 2.56 (min for S1) to 2.66 kb (max for S3), collectively contributing to a total length spanning ∼36.13 (min for S1) to 38.40 Mb (max for S4) (Fig. 1b, S3, Table S4). The gene count exceeded that of *G. lucidum* from the study by Liu *et al*. [40] (12 080) but fell below the count reported by Chen *et al*. [5] (16 113), in combination with the numbers observed in various *Ganoderma* genomes detailed by Jiang *et al*. [6] (11 267–17 553). Our findings highlight the variability in genomic gene numbers across different *Ganoderma* species and their domestication processes.

The *Ganoderma* genomes, including the five XZL varieties, exhibit a relatively compact genome, with protein-coding genes occupying a substantial portion (>70%) of each genome (Fig. 1b and S3). Consequently, repeat sequences, which typically occupy a significant portion of plant genomes, were markedly limited in XZL, accounting for only 13.19, 14.15, 13.21, 19.72 and 12.46% of the S1, S2, S3, S4 and S6 genomes, respectively. With respect to the composition of transposable elements (TE), a pattern similar to that found in plants is evident in *Ganoderma*, with long terminal repeats (LTR) also constituting a significant portion of TEs (Gypsy: 4.39–6.56%; Copia: 1.59–2.28%) (Table S5).

Furthermore, our annotation efforts extended to non-coding RNAs (ncRNAs) in the XZL genomes, revealing 162–708 rRNAs, constituting ∼0.4–1.69% of the genome, and 336–354 tRNAs, accounting for roughly 0.03–0.04% of the genome. In contrast, snRNAs were less abundant, representing only 0.01% of the genome (Tables S6–S8). Annotation of ncRNAs allows a better understanding of the structure and function of the entire genome and contributes to the future in-depth study of intracellular gene regulatory mechanisms for the development of basic biology and applied research. In summary, we conducted comprehensive gene annotation, repeat sequence annotation and ncRNA annotation on the five XZL *Ganoderma* genomes.

### Phylogenetic relationships and gene family analysis

**Fig. 2.**
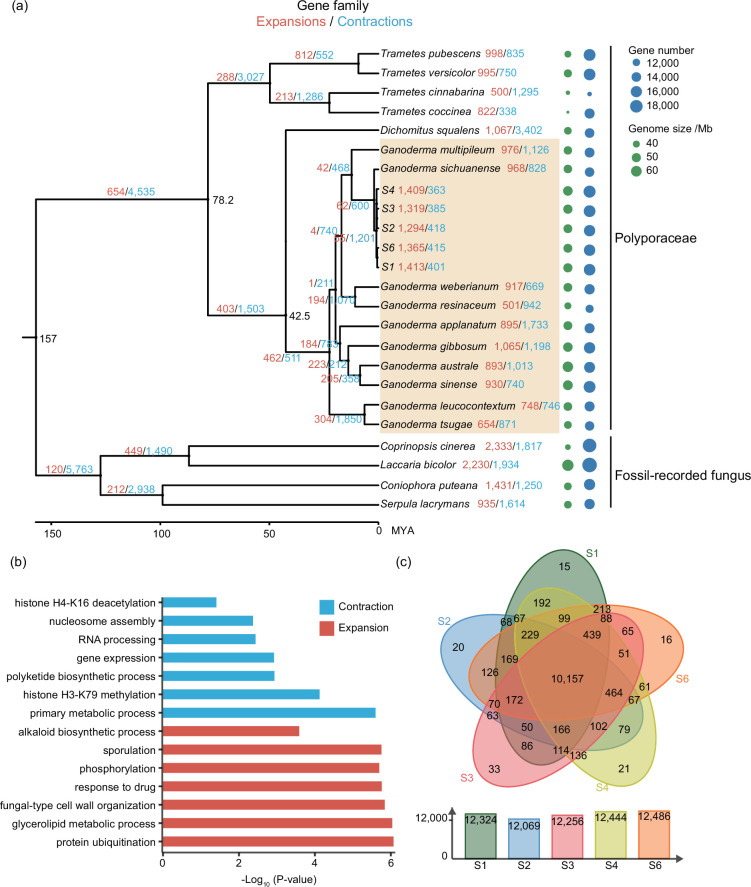
Phylogenetic tree and gene family analysis. (**a**) Phylogenetic tree depicting the relationships among 24 fungal genomes. Species highlighted with yellow backgrounds represent the *Ganoderma* species. Red and blue numbers denote the counts of expanded and contracted gene families, respectively. (**b**) GO enrichment analysis results for expanded (red bar) and contracted (blue bar) gene families in S1. (**c**) Venn diagram illustrating the number of gene families shared among the five *Ganoderma* species (S1–S6).

To determine the phylogenetic placement of the five XZL genomes as well as the time of divergence, we conducted analyses that combined 19 other fungal species: ten other species of *Ganoderma* (*G. multipileum, G. sichuanense, G. weberianum, G. resinaceum, G. applanatum, G. gibbosum, G. australe, G. sinense, G. leucocontextum* and *G. tsugae*), five other Polyporaceae species (*Trametes pubescens, Trametes versicolor, Trametes cinnabarina, Trametes coccinea* and *Dichomitus squalens*) and four fossil-recorded fungi species (*Coprinopsis cinerea, Laccaria bicolor, Coniophora puteana* and *Serpula lacrymans*), serving as outgroups (Table S9) [6,41,45]. In this comprehensive analysis, we considered 24 fungal species characterized by genome sizes ranging from 32.8 to 64.9 Mb and gene counts from 10 233 to 18 215. We began our investigation by employing OrthoFinder [28] for gene family analyses, which resulted in the identification of 16 742 gene families across the 24 fungal species, with 1216 single-copy homologous genes singled out. Subsequently, phylogenetic trees were constructed utilizing these single-copy genes. The resultant phylogenetic tree revealed that the five XZL genomes clustered within branches alongside ten other *Ganoderma* species. Among these, the XZL genomes exhibited the closest relationship with *G. sichuanense*, a *Ganoderma* species originating in southern China, sharing geographical proximity with our XZL varieties [6,46]. Moreover, our analysis also revealed that the diversification of the 15 *Ganoderma* species occurred ∼22.5 million years ago (MYA). It is notable that the phylogeny of our study aligns harmoniously with previous *Ganoderma*-related research [6], successfully elucidating the evolutionary relationship of XZL (Fig. 2a).

To explore the evolutionary history of gene families in 24 fungi, we analysed gene family expansions and contractions using CAFÉ. We found 1294–1413 gene family expansions and 363–418 family contractions in the five XZL genomes in relatively similar numbers (Fig. 2a). We further analysed the GO functional enrichment for the expanded and contracted gene families of five XZL (Fig. 2b, Tables S10–S19). Among them, expanded and contracted gene families in S1 were involved in nucleosome assembly, RNA processing, sporulation, fungal-type cell wall organization and some essential biological processes. Interestingly, we did not find gene family expansion or contraction on the internal branches of the five XZL genomes. This indicates that there are some important gene family differences between the different *Ganoderma* species, while the differences between the five XZL are comparatively low. Of the 12 376 gene families identified for XZL, a substantial 9177 (74.1%) were shared across all five XZL varieties, while gene families exclusive to each species constituted a mere fraction, accounting for less than 0.1% of the total gene families (Fig. 2c). This observation underscores the remarkably close functional similarities among the gene sets of the five XZL strains, with minimal distinctions. Hence, we determined the phylogenetic position, the age of differentiation and the expansion and contraction of the gene families of the five XZL *Ganoderma* strains, and discovered that the gene families of XZL were relatively conserved. There is a need for more in-depth studies on the important genomic differences among *Ganoderma*, especially among XZL.

### Pan-genome analysis of 15 *Ganoderma*

**Fig. 3.**
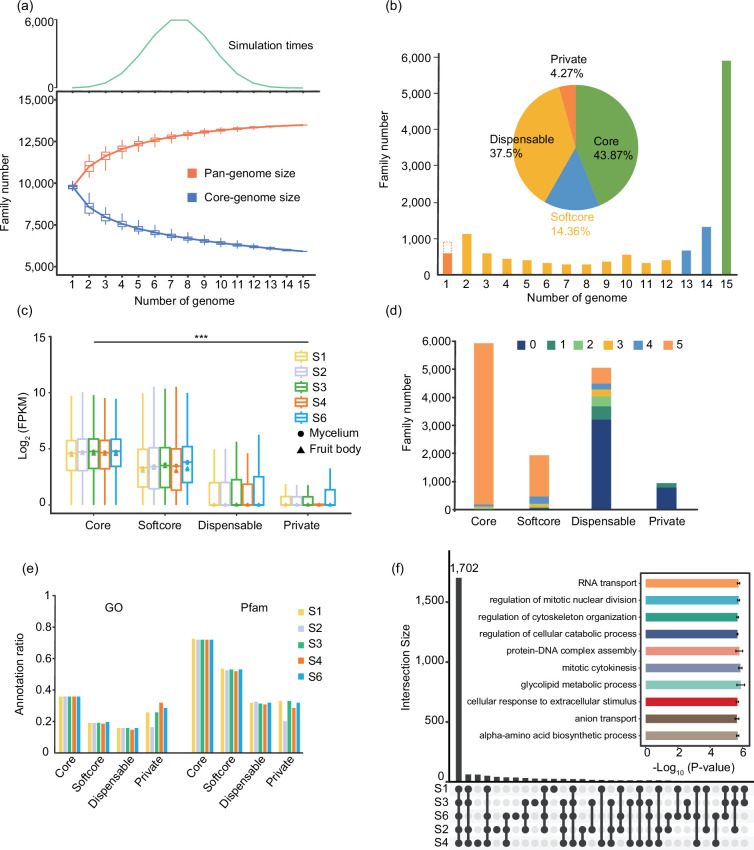
Pan- and core-genome analyses of the 15 *Ganoderma* genomes. (**a**) Variation of gene families in the pan- and core-genomes along with an increasing number of *Ganoderma* genomes. The green line in the top graph indicates the number of simulations with different numbers of genomes. (**b**) Compositions of pan- and core-gene families among the 15 *Ganoderma* genomes. The histograms and pie chart display the number and frequency of gene families among the 15 genomes, respectively. The orange bar on the left represents the number of families and genes (dashed line) that are private to each genome. (**c**) Box plot showing the expression profile of pan- and core-genes in the five XZL genomes. Different shapes indicate the medians of gene expression in different tissues of *Ganoderma*. The asterisk indicates a *P*-value of less than 0.001 among the four groups. (**d**) Expression width profile (indicated by expressed or not expressed in the five XZL genomes) of the pan- and core-gene families. Different colours represent the number of genomes. (**e**) GO and Pfam annotations for the pan- and core-genes among the five XZL genomes. (**f**) Upset plot depicting the number of core genes enriched in GO terms among the five XLZ genomes. The bar plot highlights several important biological processes, and the error lines are based on the *P*-values of the five XZL genomes.

Despite being informative, the analysis of the five XZL genomes could not entirely encapsulate the comprehensive characteristics of the entire *Ganoderma* genus. Therefore, after establishing the evolutionary relationships among the 5 XZL genomes, we embarked on a pan-genomic analysis encompassing all 15 *Ganoderma* genomes (Table S9). To this end, we used Orthofinder to categorize all genes across these genomes into 13 487 families. This count surpassed the number of gene families identified within the five XZL genomes and fell short of the gene families comprising the initial set of 24 fungal species that were examined during the study’s inception. Further evaluation of the characteristics of the pan-genome showed that, with the increase in the number of genomes, the number of total gene families increased but tended to flatten out to a certain extent (Fig. 3a). This observation suggests that the pan-genome, constructed using these 15 representatives *Ganoderma* varieties, effectively encapsulates a substantial portion of the genetic information inherent to *Ganoderma* spp.

To gain a better understanding of the gene composition characteristics within the *Ganoderma* pan-genome and to elucidate differences in the frequency of gene occurrence among these 15 materials, we classified gene families into four distinct categories. Among these, the core families, encompassing genes present in all 15 species, amounted to 5917 families, constituting 43.87% of the total. Softcore families, observed in 13–14 species, were represented by 1937 families, accounting for 14.36%. Families identified in 2–12 species were designated as dispensable families, totalling 5058 families or 37.5%. Finally, gene families detected in only one species were catalogued as private gene families, amounting to 575 families, which was equivalent to 4.26% of the total. Furthermore, we identified 368 independent genes in our five XZL genomes that did not fall under the gene family analysis; these genes were collectively analysed as independent private gene families (Fig. 3b). Furthermore, we examined the distribution patterns of different gene families across the *Ganoderma* genome (Fig. S4). Our findings showed that the *Ganoderma* genome exhibited distinct characteristics in contrast to plant genomes. It featured a smaller genome size with fewer repeat sequences, and different gene families displayed a relatively even distribution throughout the genome, lacking the specific distribution patterns observed in plants [47,48]. To summarize, our analysis revealed the rich diversity of gene families in the *Ganoderma* pan-genome and emphasized the importance of core families. This suggests that, in spite of genetic variation between cultivated strains in different *Ganoderma* genomes, most genes remain relatively stable, preserving their essential gene functions.

To delve deeper into the distinct characteristics of various gene families, we closely examined the expression patterns of different gene types within XZL *Ganoderma*. We observed a consistent expression level across the same categories of gene families. Notably, the expression levels exhibited a gradual decrease from core to softcore and then to dispensable and private gene families. Intriguingly, we noted a similar expression pattern between the mycelium and fruit bodies in *Ganoderma* (Fig. 3c). Hence, as the individual genetic differences in the *Ganoderma* genome diversity increase, the fluctuation in gene expression also increases. In addition, when we assessed the breadth of expression across the different XZL genomes, core genes exhibited the widest expression profile (Fig. 3d), emphasizing their stable and extensive role in gene expression within *Ganoderma*. Gene annotation results using GO and Pfam revealed a diminishing proportion of genes that could be annotated as we moved from core genes to softcore, dispensable and private genes (Fig. 3e). Moreover, our analysis of the functional annotation of the core genes of each XZL species revealed that the five XZLs were annotated to many common entries, and all of them were involved in RNA transport, mitotic cytokinesis, regulation of cellular catabolic processes, and some important biological processes of species development (Fig. 3f). In summary, our findings highlight the evolutionary conservation and central role of core genes in *Ganoderma*, aligning with insights from studies in the realm of plants [11,49]. These core genes emerge as key players in the growth and development of *Ganoderma*.

### Genetics structure analysis for five XZL genomes

**Fig. 4.**
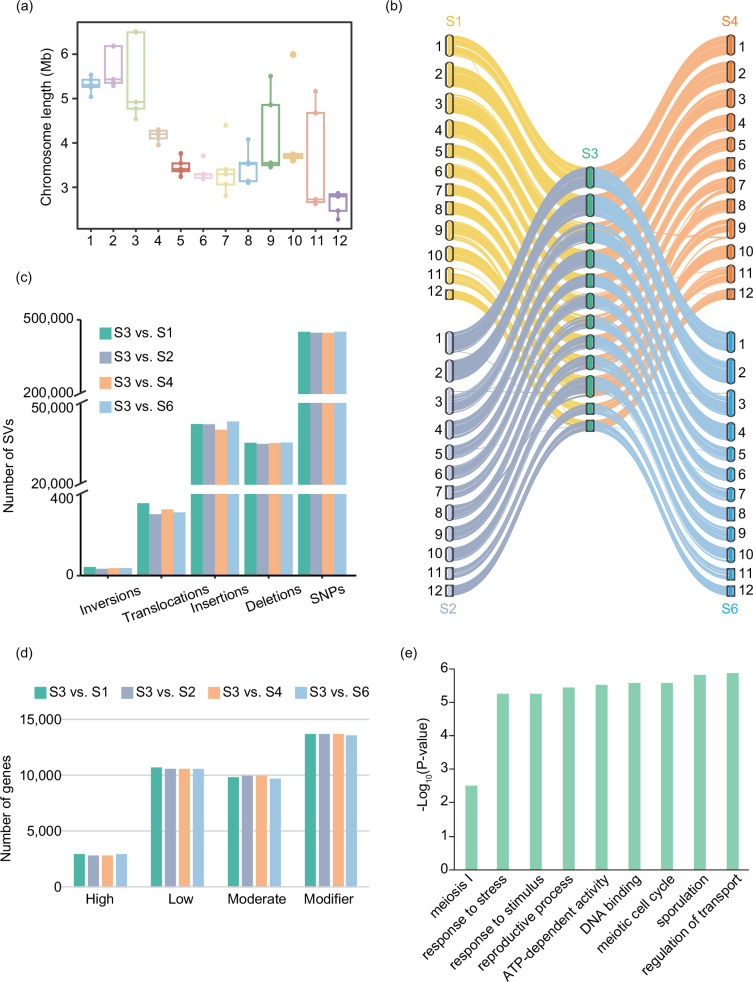
Genome structure analysis of the five XZL genomes. (**a**) Box diagram showing the chromosome lengths of the five XZL genomes. (**b**) Gene collinearity analysis of S1, S2, S4 and S6 compared to S3. (**c**) Bar plot showing the number of SVs. (**d**) Bar plots displaying the number of genes affected by different categories of SVs. (**e**) GO enrichment analysis of genes associated with high-impact SVs for S3.

To further reveal the structural characteristics embedded within the five XZL genomes, we performed a comparative genomic analysis of these genomes. Our exploration showed variances in chromosome length among the five XZL genomes (Fig. 4a), a phenomenon also observed in previously released *Ganoderma* genomes [6]. Additionally, we noted differences in chromosome numbers among genomes of different *Ganoderma* species [6]. Afterwards, we engaged in genome-wide collinearity analysis of the five XZL genomes, uncovering the majority of sequences that exhibited collinearity among different genomes. However, we also identified the presence of SVs (Fig. 4b). As a new variety of *Ganoderma*, S3 combines a variety of excellent characteristics. To delve deeper into these SVs, we categorized the five XZL genomes into four groups using S3 as the anchor (S3 vs. S1, S3 vs. S2, S3 vs. S4, and S3 vs. S6) for detailed structural variant analysis. This analysis revealed the presence of 428 493, 425 285, 424 410 and 429 002 SNPs and 78 158, 77 591, 75 950 and 79 167 SVs respectively, in the genomes of the other four varieties compared to S3 (Fig. 4c).

Depending on their location within the gene, SVs can exert varying degrees of influence on genes. We categorized SVs into four groups based on their impact on genes: high, moderate, low and modifier. The number of SVs in the four groups was relatively close, resulting in a similar number of genes affected by SVs (Fig. 4d). The genes affected by high degrees of SVs were mainly involved in critical biological functions, such as response to stress, response to stimulus, DNA binding, meiotic cell cycle and sporulation (Fig. 4e). Our SV analysis sheds light on the genomic differences within the five XZL genomes. The key genes that received SV effects were characterized for their involvement in important biological functions, and these SVs may have an impact on the meiotic process as well as spore formation in *Ganoderma*.

### Impact of SVs on XZL spore synthesis genes in different strains

**Fig. 5.**
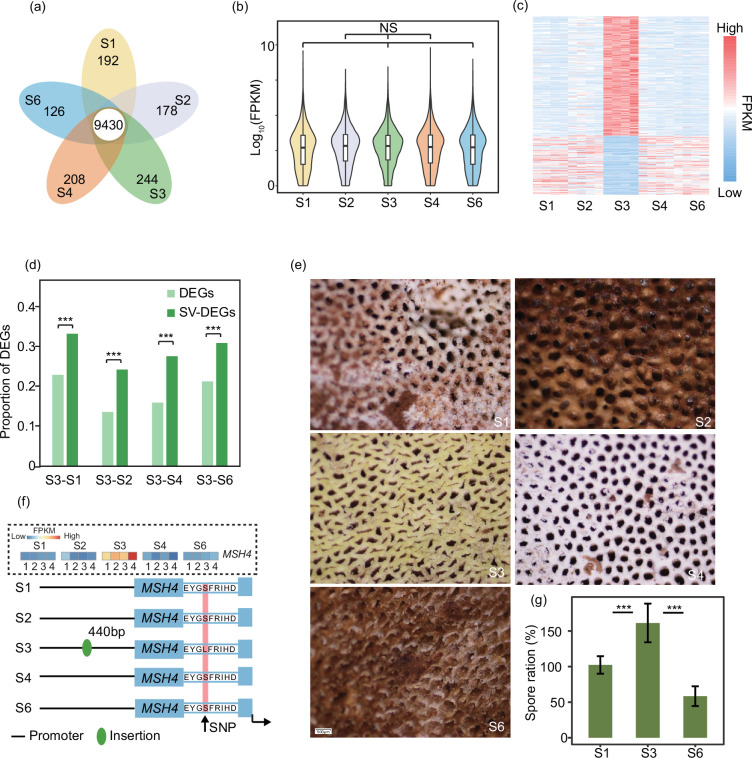
Impact of SV on homologous gene expression in *Ganoderma*. (**a**) Number of homologous genes in the five XZL genomes. (**b**) Violin plot showing the expression pattern of homologous genes in the five XZL genomes. (**c**) Heatmap showing gene patterns that are differentially expressed in S3 versus other XZLs. (**d**) Histogram showing the proportion of DEGs and SVs-DEGs in homologous genes. ***P-value <0.001. (**e**) Spore aperture photos of five XZLs of the same size. (**f**) Sketch of the *MSH4* gene in five XZLs. (**g**) Bar plot showing the ratio of spore content to dry weight of the fruiting body for S1, S3, and S6. ***P-value=<0.001 for the t-test.

To gain deeper insights into the impact of SVs on gene expression, our initial investigation centred on the overall expression levels of homologous genes found in the five XZL genomes, totalling 9430 genes, with no significant differences in expression levels among these five XZL varieties (Fig. 5a, b). However, some genes were still differentially expressed in the other four XZL varieties compared to S3 (Fig. 5c). Building on our previous study, which found that SVs in genes affect gene expression, we investigated the differential expression of homologous pairs of genes in which SVs occurred. We noted that the proportion of DEGs in homologous genes with SVs was notably higher than the proportion of DEGs in all genes (Fig. 5d). This observation suggests that SVs, particularly within the gene regions, have a notable influence on gene expression, resulting in a higher likelihood of differential gene expression among homologous genes with SVs.

Spores act both as the reproductive cells of *Ganoderma* and as a valuable source of medicinal compounds, including polysaccharides, triterpenoids and bioactive substances, known for their immune-modulating, antioxidant, anti-inflammatory, and antitumour properties [50,51]. S3 had a greater number of spore apertures per unit area (Fig. 5e), making its spore count higher than that of the other species. The previous key genes for S3 expansion and contraction, as well as SV effects, were similarly enriched for important meiosis and spore development key pathways. Previous research [52,53] has shown that *MSH4* is a key factor affecting fungal spore production. Interestingly, we found that among the key genes affecting spore development, *MSH4* in S3 had some of the same SVs as the other four species (Fig. 5f). The presence of these SVs may influence the expression of *MSH4*, making the expression level of *MSH4* in S3 much higher than in the other four varieties (Fig. 5f). Our findings may be the main reason for the dramatic increase in spore production in S3. We determined the spore content of S1 and S3 by experimental means, and the experimental results corroborated our conjectures (Fig. 5g). In brief, our study suggests that SVs in *MSH4* play a pivotal role in influencing the expression of key genes, ultimately impacting sporulation yield. These findings provide invaluable theoretical insights that contribute to the cultivation and enhancement of desirable traits in *Ganoderma*.

## Discussion

A medicinal fungus widely used in traditional Chinese medicine, *G. lucidum* belongs to the large genome species of fungi. In recent years, significant strides have been witnessed in *Ganoderma* genome research [5,6]. Delving into the genomes of distinct *Ganoderma* species not only elucidates their genetic diversity but also bolsters efforts to select and cultivate high-yield, high-quality *Ganoderma* varieties while preserving the diversity of wild *Ganoderma* populations. In the present study, we comprehensively assembled high-quality reference genomes and comprehensive annotations for five XZL *Ganoderma* species native to Fujian, China. This endeavour harnessed the power of three generations of sequencing technologies: PacBio HiFi, Hi-C and second-generation Illumina sequencing (Fig. 1b). In particular, our assessment analysis revealed an exceptionally high level of genome integrity, particularly in terms of gene annotation integrity (Table 1). High-quality reference genomes can significantly enhance our understanding of the genomic structure, gene functions and metabolic pathways of *Ganoderma*. This deeper insight not only advances research into its medicinal properties and biological characteristics but also serves as a valuable resource for genetic improvement. With this genomic information, targeted breeding programmes can be developed to cultivate more potent medicinal varieties or strains of *Ganoderma* with desirable traits.

Based on genome-wide information from five XZL genomes, we performed phylogenetic analyses with 19 other fungal species. The time of divergence between species, extrapolated using the four fossils as outgroups, remained consistent with the record [6]. We noted a potential correlation between the evolutionary distance of *Ganoderma* and its geographic location, a phenomenon highlighted by the proximity of XZL and *G. sichuanense* (Fig. 2a). Previous studies have posited that the north–south differentiation of *Ganoderma* may be attributable to climatic and topographical factors [6,54], warranting further investigations into the specifics of this differentiation. By comparing the genomes of diverse *Ganoderma* species, we can unveil their evolutionary history and relationships, shedding light on the genetic affinities among distinct *Ganoderma* species and their distribution across different geographical regions. Comparing the genome of *Ganoderma* with those of other fungi can provide valuable insights into its evolutionary position and ecological adaptations within the fungal kingdom. This comparative analysis offers robust support for studies in fungal ecology and phylogenetics.

In this study, a pan-genomic study was conducted on five XZL and ten other species of *Ganoderma* species. The *Ganoderma* genomes exhibited a relatively uniform size and gene count (Fig. 2a). A significant proportion of the genes within these genomes were core or softcore genes, occupying pivotal roles in developmental processes (Fig. 3b). It is of note that our investigation revealed noteworthy genetic variations among the five XZL genomes. We stratified the SVs based on their impact on genes, revealing that high-impact SVs pertained to genes involved in spore development and meiotic cell cycle processes (Fig. 4e). S3 is a new variety of XZL with many excellent strains. Its high spore yield and many spore apertures may allow it to grow in large numbers locally in the future (Fig. 5e, g). We found that its high spore content may be due to the high expression of the key *MSH4* gene. The reason for this high expression is the presence of SVs in the promoter region or gene body (Fig. 5f). *MSH4* has been shown to play a crucial positive regulatory role in *Ganoderma* spore development [52]. The homologs *stpp1* [55] and *poMSH4* [53] of *MSH4* are responsible for spore formation in *Pleurotus pulmonarius* and *Pleurotus ostreatus*, respectively.

To summarize, this study resulted in the high-quality assembly of five XZL genomes, representing the inaugural pan-genomic exploration of *Ganoderma*. Not only does this enrich the data foundation for probing the molecular mechanisms underpinning *Ganoderma* but also provides a profound understanding of the biological underpinnings of this medicinal fungus. Such insights hold promise for optimizing its production and applications and advancing our comprehension of its therapeutic utility in traditional Chinese medicine. It also provides a scientific foundation for the standardized cultivation, management and quality control of *Ganoderma*, helping to drive the modernization and large-scale development of the *Ganoderma* industry.

## supplementary material

10.1099/mgen.0.001328 Uncited Supplementary Material 1.

10.1099/mgen.0.001328 Uncited Supplementary Material 2.

10.1099/mgen.0.001328 Uncited Supplementary Material 3.

## Abbreviations

DEG: differentially expressed gene
GO: gene ontology
GWH: genome warehouse
LTR: long terminal repeats
MYA: million years ago
ncRNAs: non-coding RNAs
PDA: potato dextrose agar
SRA: sequence read archive
SV: structural variation
TE: transposable element
XZL: Xianzhilou
