# Supplementary material for: Pan-genomic characterization and structural variant analysis reveal insights into spore development and species diversity in Ganoderma
Source: Microb Genom. 2024 Nov 20;10(11):001328. doi: 10.1099/mgen.0.001328 (PMC11897173; doi:10.1099/mgen.0.001328)
Supplement: Uncited Supplementary Material 1. [file mgen-10-01328-s001.pdf]

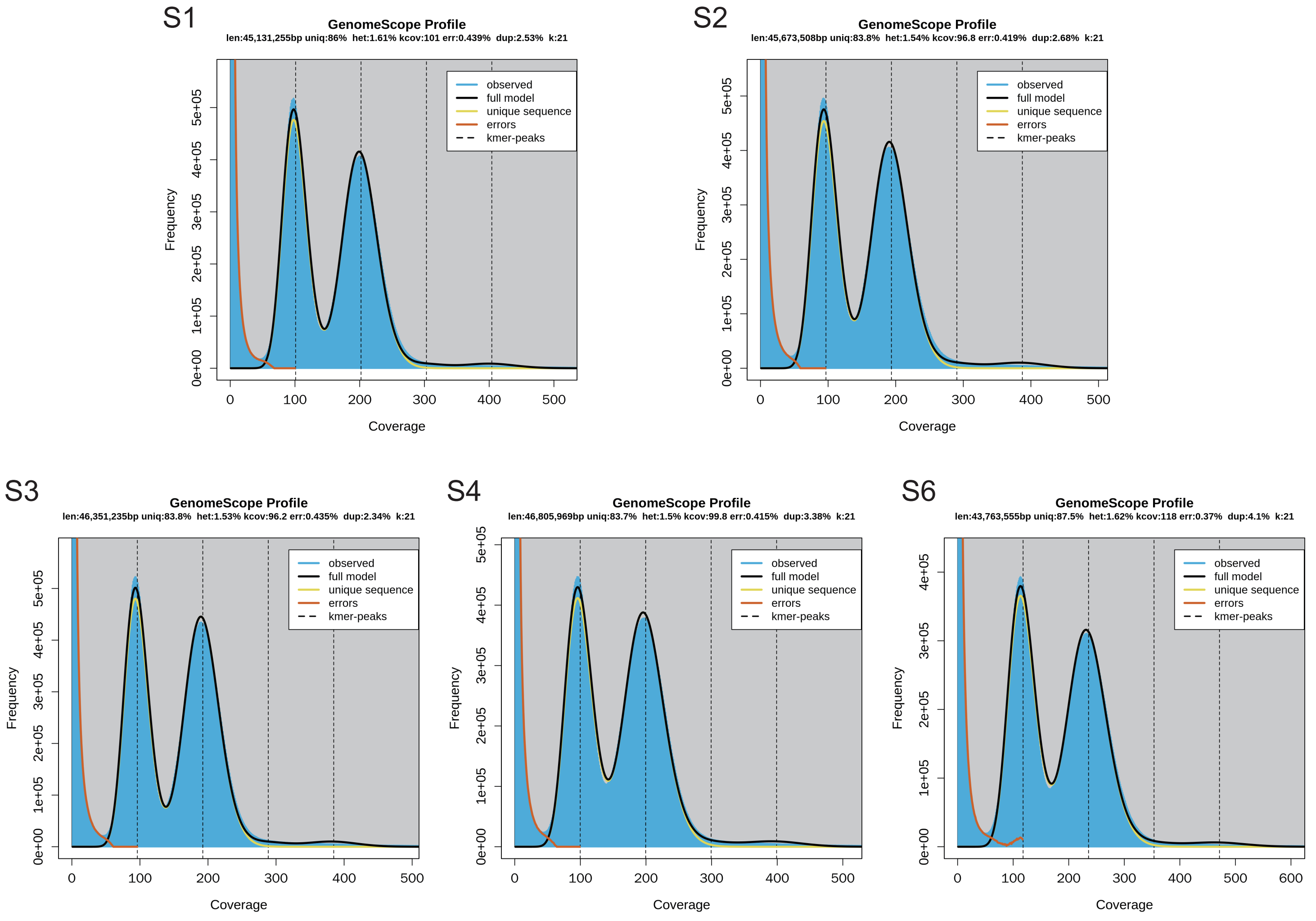

Fig. S1. Genome survey of 5 XZL on the distribution of 21-mer.

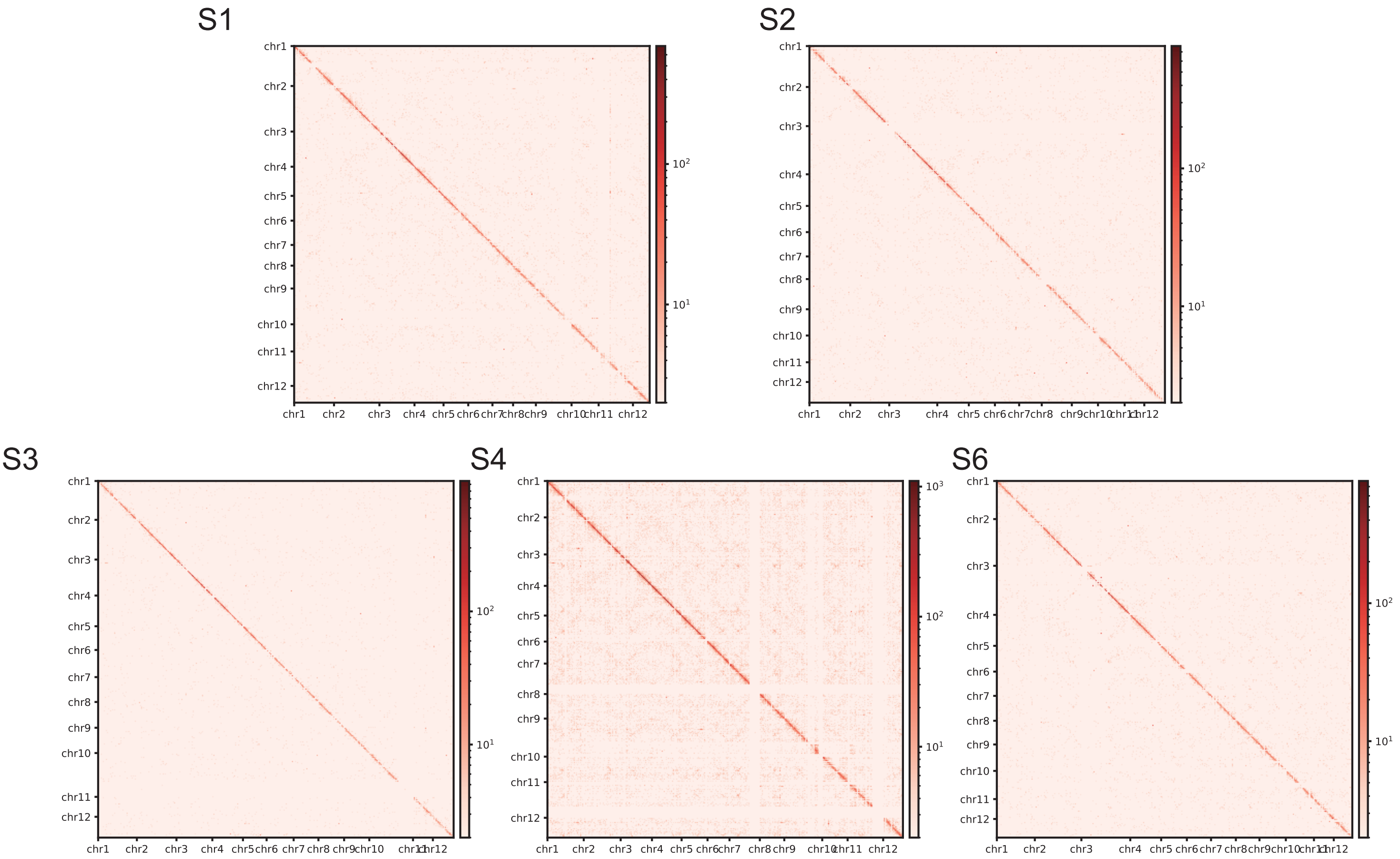

Fig. S2. Hi-C heatmap of 5 XZL genomes.

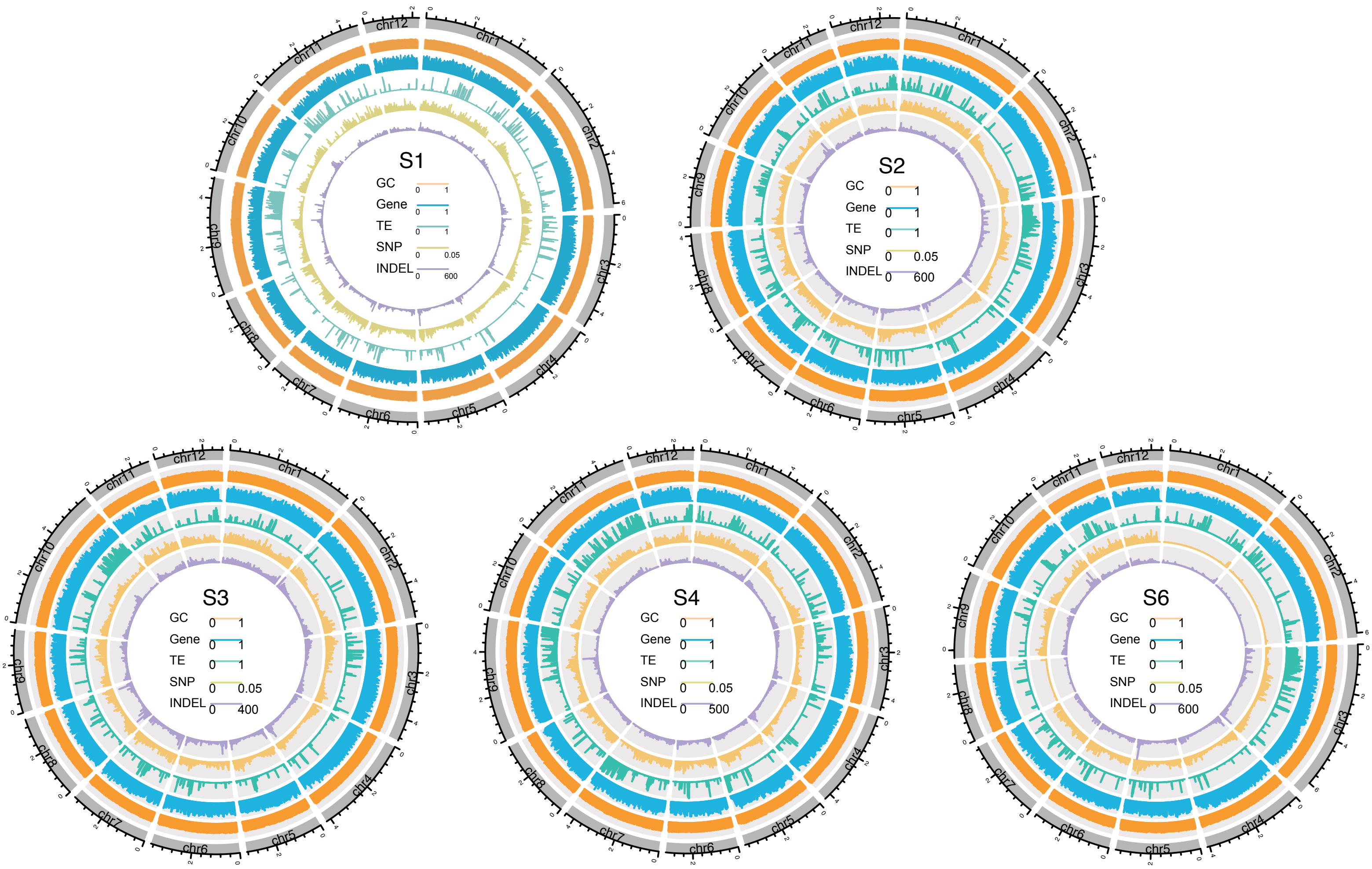

Fig. S3. Circos plot showing the annotation of 5 XZL genomes.

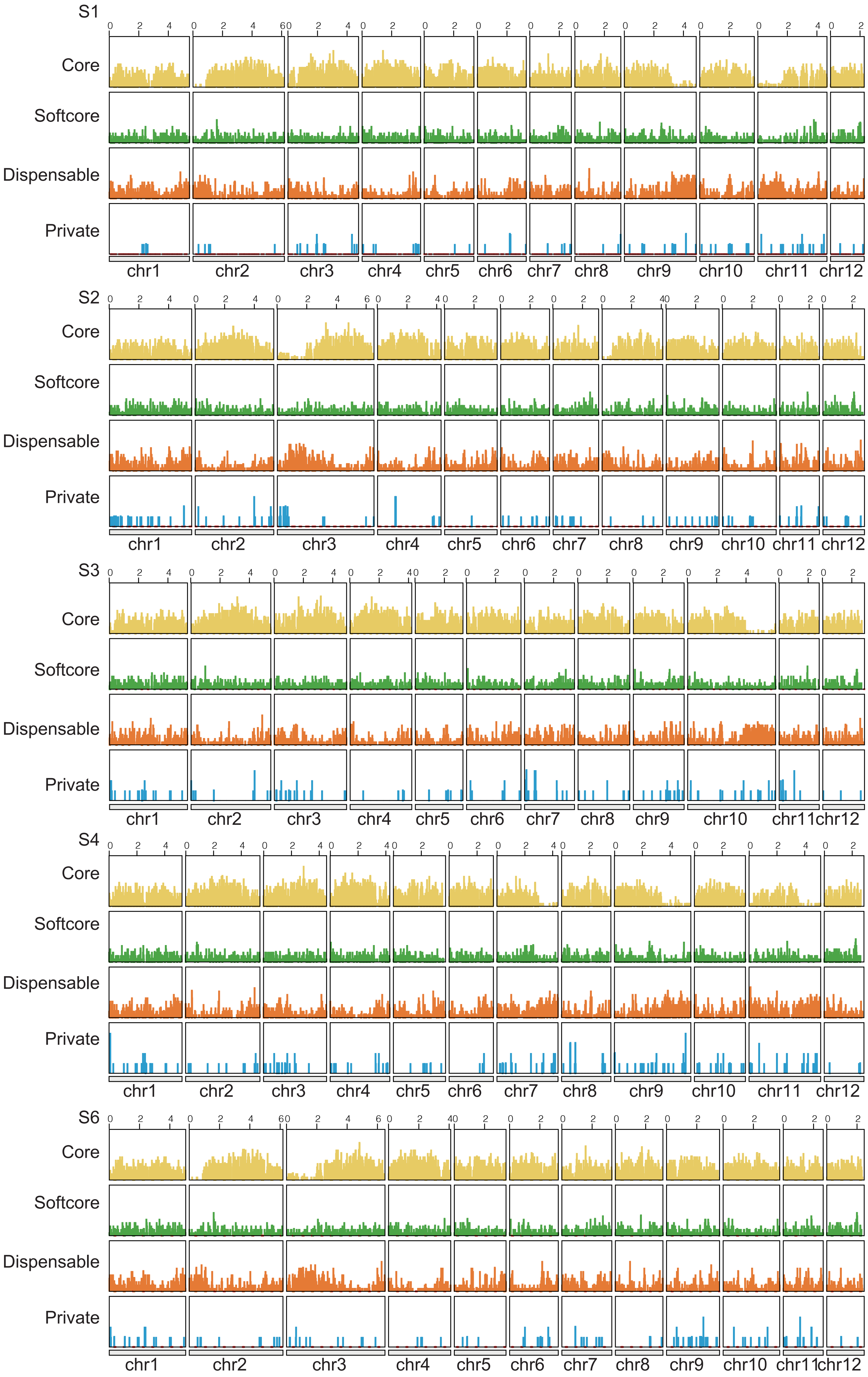

Fig. S4. Distribution of different gene families in 5 XZL genomes.
