# Supplementary material for: Pan-genomic characterization and structural variant analysis reveal insights into spore development and species diversity in Ganoderma
Source: Microb Genom. 2024 Nov 20;10(11):001328. doi: 10.1099/mgen.0.001328 (PMC11897173; doi:10.1099/mgen.0.001328)
Supplement: Uncited Supplementary Material 2. [file mgen-10-01328-s002.pdf]

Table S1. Statistics of Illumina sequencing data.

	S1	S2	S3	S4	S6
Total reads	89,502,942	90,859,188	92,966,392	92,854,396	108,741,618
Total bases/bp	13,425,441,300	13,628,878,200	13,944,958,800	13,928,159,400	16,311,242,700
Read length/bp	150:150	150:150	150:150	150:150	150:150
GC/%	53.07;52.88	51.52;51.34	51.43;51.17	52.67;52.43	50.41;50.45
Q20/%	98.42;97.63	98.50;97.64	98.44;97.56	98.54;97.69	98.58;97.75
Q30/%	94.55;92.92	94.82;93.00	94.60;92.71	94.96;93.17	95.21;92.72

Table S2. Statistics of PacBio sequencing data.

	S1	S2	S3	S4	S6
Total Reads	250,059	296,132	259,594	227,390	268,861
Total Bases/bp	4,153,082,434	5,065,045,334	4,370,727,226	3,794,816,624	4,451,867,159
Coverage	92	111	94	81	102
Max read/bp	39,332	47,359	32,330	38,537	42,233
Median read/bp	15,979	16,402	16,183	16,068	15,981
Average read/bp	16,608	17,104	16,837	16,689	16,558
N50	16,387	16,918	16,636	16,475	16,364
Min read/bp	708	1,052	410	693	263

Table S3. Statistics of Hi-C sequencing data

Sample	Length/bp	Q20/%	Q30/%	GC/%	Total Reads	Total Bases
S1	150;150	96.55;97.18	89.93;91.27	56.50;56.37	123,636,988	18,545,548,200
S3	150;150	96.67;96.94	90.22;90.62	56.93;56.83	116,356,610	17,453,491,500
S4	150;150	96.68;96.96	90.23;90.71	55.77;55.69	118,564,040	17,784,606,000

Table S4. Gene annotation of 5 XZL genomes.

	S1	S2	S3	S4	S6
Gene count	14,140	13,897	14,362	14,779	13,959
Total gene length /bp	36,127,837	36,271,825	38,143,916	38,404,058	36,588,641
Average gene length /bp	2555.01	2610.05	2655.89	2598.56	2621.15
Gene proportion in genome /%	72.47	74.76	75.55	72.37	75.25
Mean exons number	6.19	6.18	6.22	6.14	6.27
Mean CDS length /bp	235.46	235.77	235.77	239.54	231.90
Mean intron length /bp	509.47	530.63	530.72	520.81	521.78

Table S5. Repeat sequence annotation of 5 XZL genomes.

	S1	S2	S3	S4	S6
Total repeat sequence/%	13.19	14.15	13.21	19.72	12.46
Gypsy/%	6.46	6.56	4.39	5.46	4.55
Copia/%	1.62	2.28	1.59	2.25	1.94
DNA/%	0.89	0.48	1.58	3.23	1.33
LINE/%	0.00	0.02	0.08	1.83	0.02
OTHER/%	4.22	4.81	5.57	6.95	4.62

Table S6. rRNA annotation of 5 XZL genomes.

rRNA	S1	S2	S3	S4	S6
Number	594	163	708	624	595
Total length/bp	829,886	193,405	851,076	720,054	712,979
Proportion in genome/%	1.66	0.40	1.69	1.36	1.47

Table S7. tRNA annotation of 5 XZL genomes.

tRNA	S1	S2	S3	S4	S6
Number	352	337	336	354	337
Total length/bp	18,092	17,048	16,906	18,075	16,941
Proportion in genome/%	0.04	0.04	0.03	0.03	0.03

Table S8. snRNA annotation of 5 XZL genomes.

snRNA	S1	S2	S3	S4	S6
Number	21	21	21	21	21
Total length/bp	2,746	2,743	2,742	2,742	2,747
Proportion in genome/%	0.01	0.01	0.01	0.01	0.01
